# First Clinical Use of a Novel AI-Based Imaging Tool to Enhance CTO PCI Planning

**DOI:** 10.1016/j.jaccas.2025.106311

**Published:** 2025-12-04

**Authors:** Hassan Saleh, Jaikirshan Khatri

**Affiliations:** New York Presbyterian/Weill Cornell Medical Center, New York, New York, USA

**Keywords:** chronic total occlusion, coronary angiography, CTO, enhanced angiogram, imaging, PCI, percutaneous coronary intervention, spatiotemporal enhancement processing

## Abstract

**Background:**

Chronic total occlusion (CTO) interventions are frequently limited by incomplete angiographic information. We report the use of a novel artificial intelligence (AI)–based spatiotemporal enhancement processing (STEP) software (AngioWave Imaging), which identified both an antegrade crossing pathway and an epicardial collateral, neither of which was apparent on the unprocessed angiogram. These insights guided the procedural strategy, including the decision to attempt parallel wiring, thereby contributing to procedural success.

**Case Summary:**

A 59-year-old man presented with non–ST-segment elevation myocardial infarction, with the diagnostic angiogram revealing a culprit left anterior descending artery lesion and a circumflex CTO. After undergoing culprit vessel percutaneous coronary intervention (PCI), he was referred for CTO PCI. Angiographic assessment of the circumflex CTO was limited. Enhancement of the angiogram was performed using the STEP software, which revealed a microchannel and an epicardial collateral, both not evident on the unprocessed angiogram. On the basis of this information, we used a successful parallel wiring strategy.

**Discussion:**

This case report presents the first clinical use of AI-based STEP image enhancement in PCI. Conventional angiography may fail to adequately delineate lesion architecture and collateral circulation, both of which are critical for CTO case planning. In this patient, AI-based enhancement identified an antegrade microchannel that enabled parallel wiring and avoided premature conversion to a retrograde strategy in a vessel not suitable for antegrade dissection and re-entry. STEP-enhanced angiograms provided actionable procedural guidance beyond standard imaging, underscoring their potential role in complex interventions.

**Take-Home Messages:**

Understand how STEP-based image-processing platforms can augment strategy selection in CTO PCI and reduce procedural time, radiation, and contrast exposure.

## Graphical Abstract

**Figure:**
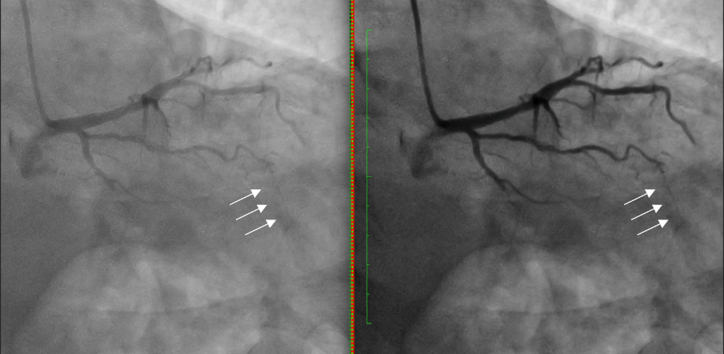

## History of Presentation

A 59-year-old gentleman presented to the hospital with 1 week of stuttering chest pain. The initial electrocardiogram revealed sinus rhythm with a rate of 78 beats/min and nonspecific ST-segment changes; high-sensitivity troponin values of 61, 68, and 61 ng/L; and a creatinine level of 0.89 mg/dL. A transthoracic echocardiogram showed preserved left ventricular ejection fraction (55%-60% by visual inspection) with no regional wall motion abnormalities. His TIMI and Global Registry of Acute Coronary Events scores were 3 and 90, respectively. He received 324 mg of aspirin, was started on a heparin infusion, and underwent coronary angiography the following day.

**Take-Home Message**

• Understand how spatiotemporal enhancement processing–based image-processing platforms can augment strategy selection in chronic total occlusion percutaneous coronary intervention and reduce procedural time, radiation, and contrast exposure.

## Past Medical History

The patient had a history of coronary artery disease (coronary artery calcium score 131), hypertension, diabetes mellitus, obesity, renal cell carcinoma status post–partial nephrectomy, and prostate cancer status postprostatectomy (while taking leuprolide).

## Investigations

### Initial angiography

Initial angiography revealed a culprit mid–left anterior descending artery lesion and a distal circumflex chronic total occlusion (CTO). Intervention of the culprit lesion was undertaken with the placement of a 2.5 × 28 mm XIENCE Skypoint stent (Abbott) after dilation with a 3.0 × 15 mm NC Euphora balloon (Medtronic) with a minimum stent area of 5.3 mm^2^ as measured by intravascular ultrasound (IVUS) (OPTICROSS HD IVUS catheter, Boston Scientific).

**Figure 1:**
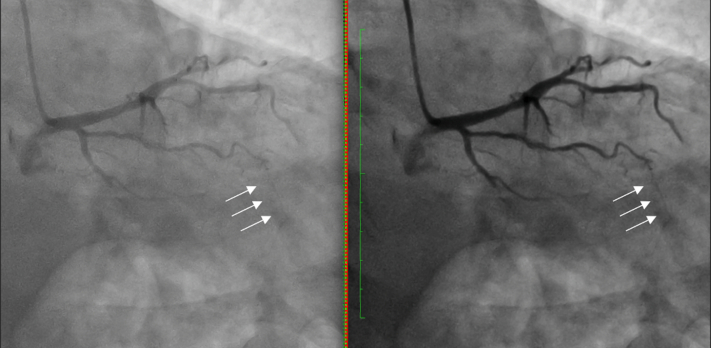
Ipsilateral Collateral Arising From the First Obtuse Marginal Highlighted With the White Arrow (RAO 13°, CAU 36°; Unprocessed on Left, Processed on Right) CAU = caudal; RAO = right anterior oblique.

Given his presentation with acute coronary syndrome, we decided to proceed with CTO percutaneous coronary intervention (PCI) of the circumflex for complete revascularization before discharge. After review of the angiogram, it was unclear whether 1) there was an antegrade channel, 2) the origin of collateral circulation, or 3) the source of the contralateral collateral circulation (Videos 1 to 4). Because of this ambiguity, we processed the angiogram using a novel artificial intelligence–based spatiotemporal enhancement processing (STEP) software (AngioWave Imaging) (Figure 1, Videos 1 to 4). On the basis of the processed angiogram, we concluded that the ipsilateral collateral circulation was arising from the obtuse marginal and that the contralateral collateral circulation was not an interventional collateral.

## Management

### CTO PCI

A 5/6 Glidesheath Slender kit (Terumo) was placed in the right radial artery and an 8-F × 45 cm Super Arrow Flewx Sheath (Teleflex) in the right common femoral artery. Anticoagulation was achieved with intravenous unfractionated heparin (activated clotting time >300 seconds throughout). A 6-F Launcher 3D Right catheter (Medtronic) was used to engage the right coronary artery, followed by the placement of a Sion Blue guidewire (Asahi Intecc). An 8-F Launcher Extra Backup 3.5 catheter (Medtronic) was used to engage the left main coronary artery.

**Figure 2:**
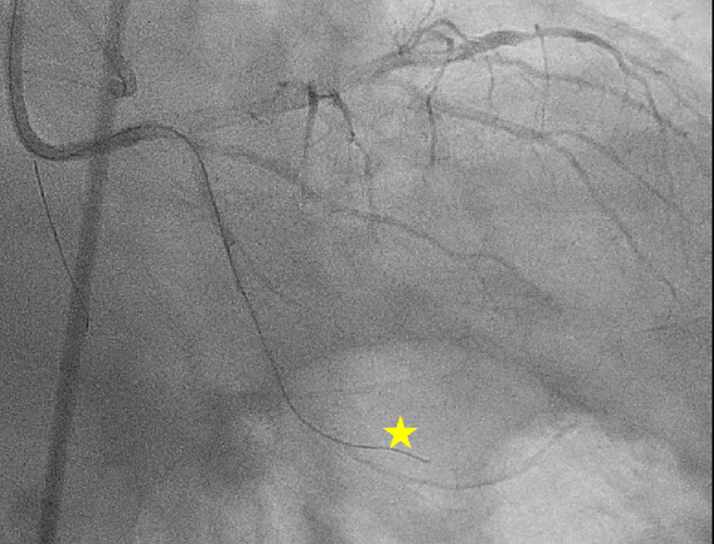
Ipsilateral Injection Demonstrating the Fielder XT-A (Yellow Star) in the Extraplaque Space (RAO 11°, CAU 31°) Abbreviations as in Figure 1.

**Figure 3:**
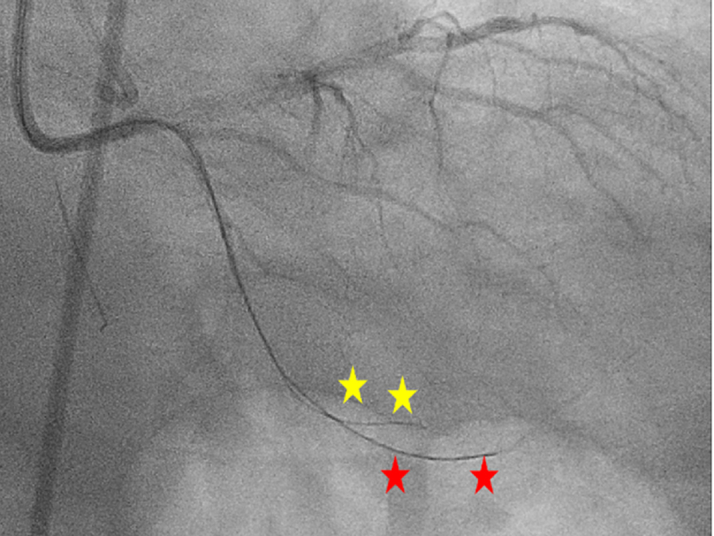
Ipsilateral Injection Demonstrating the Gaia Next 1 (Red Stars) in the Distal True Lumen and the Fielder XT-A (Yellow Stars) in the Extraplaque Space (RAO 11°, CAU 31°) Abbreviations as in Figure 1.

After dual angiography, a Sion Blue guidewire in a 135 cm Mamba Flex microcatheter (Boston Scientific) was positioned at the proximal cap of the CTO. The Sion Blue guidewire was exchanged for a Fielder XT-A guidewire (Asahi Intecc), which tracked extraplaque (Figure 2). Given that we had observed a microchannel on the processed angiogram, we elected to proceed with parallel wiring rather than switch to an alternate strategy. The Mamba Flex microcatheter was then trapped with a TrapIT balloon (Interventional Medical Device Solutions), loaded with a Sion Blue guidewire, and advanced to the proximal cap. The Sion Blue guidewire was exchanged for a Gaia Next 1 (Asahi Intecc), which was maneuvered to track above the extraplaque Fielder XT-A guidewire and successfully crossed the CTO into the distal true lumen. The true lumen was confirmed using orthogonal angiographic projections and ipsilateral injection (Figure 3). The Mamba Flex microcatheter was then tracked over the Gaia Next 1, after which the wire was removed and a phasic waveform was transduced off the microcatheter. A RUNTHROUGH NS Extra Floppy (Terumo) was advanced, and the microcatheter was removed using the TrapIT balloon.

Predilation with a 2.0 × 30 mm Euphora balloon was performed, followed by IVUS, revealing a distal reference diameter of 2.5 mm and a proximal reference diameter of 3.0 mm. Serial dilations were performed with a 2.5 × 15 mm NC Euphora balloon at 20 atm. A 2.5 × 48 mm XIENCE Skypoint everolimus-eluting stent was then deployed from the distal circumflex artery across the CTO segment to the mid–circumflex artery. Postdilation was performed with a 2.5 × 15 mm NC Euphora balloon in the distal and mid segments at 20 atm and with a 3.0 × 15 mm NC Euphora balloon in the proximal third of the stent at 20 atm. Repeat IVUS confirmed adequate expansion, apposition, and no edge dissection. Final angiography demonstrated TIMI flow grade 3 without complications (Video 5).

The total fluoroscopic and procedural times were 24.4 and 80 minutes, respectively; air kerma was 665 mGy; the dose-area product was 52.6 Gy·cm^2^; and 160 mL of Omnipaque 350 (GE Healthcare) was administered.

## Outcome and Follow-Up

The patient was discharged the following day with a prescription of aspirin 81 mg once daily and ticagrelor 90 mg twice daily.

## Discussion

CTO lesions are identified in 15% to 20% of patients undergoing coronary angiography.^1^, ^2^, ^3^, ^4^ Despite the availability of advanced tools and steadily improving procedural outcomes, only 10% of CTO lesions undergo PCI, accounting for <5% of overall PCI volume.^2^^,^^5^, ^6^, ^7^, ^8^ One key barrier is the variability in operator interpretation of angiographic complexity, which significantly influences strategy selection and success.^2^^,^^6^^,^^9^

The J-CTO (Multicenter CTO Registry of Japan) score remains the most widely used grading system to estimate the probability of antegrade wire crossing.^9^ However, the reproducibility of J-CTO and other lesion complexity scores is limited by angiographic image quality and the subjective nature of collateral assessment. Computed tomography angiography (CTA) has emerged as a complementary planning tool, and CTA-derived J-CTO scores have been shown to better predict procedural success compared with angiography-derived scores.^10^ Yet, CTA adds additional testing, contrast exposure, and logistical complexity.

Recent advances in artificial intelligence and machine learning have further highlighted the potential of imaging enhancement in cardiovascular care. Deep learning models have demonstrated accurate detection of obstructive stenosis on CTA and robust quantification of epicardial and thoracic adipose tissues.^11^^,^^12^ Building on these concepts, STEP is an emerging tool that improves the signal-to-noise ratio, clarifying vessel architecture and collateral channels that may not be visualized on standard cineangiography.

The AngioWave STEP model was trained on anonymized angiographic cine data obtained from the Cardiovascular Core Lab at MedStar Washington Hospital Center from a population whose baseline characteristics were representative of a typical catheterization laboratory population. A total of 120 angiographic cines were annotated by readers to delineate vessel pixels, creating detailed masks of the vasculature. Ground truth annotations were used as target outputs during supervised training, with the network learning to minimize the differences between predicted and annotated masks. The STEP model was then prospectively validated using annotated angiograms not previously seen by the algorithm. STEP was shown to improve an array of objective computations of image quality as well as to pass an extensive task-based reader study verifying improved vessel visibility and lack of artifacts. Currently, processing is done offline. The process involves downloading the DICOM file and selecting cines for processing, which are then uploaded on a web browser and processed; a typical cine is processed in 30 to 60 seconds, downloaded, and then opened in a DICOM viewer.

In our case, STEP enhanced the visibility of an antegrade path through a circumflex CTO and identified an ipsilateral collateral, both of which were not clearly evident on the unprocessed angiogram. Guided by this information, we successfully crossed the CTO using parallel wiring rather than prematurely converting to a retrograde strategy in a vessel not suitable for antegrade dissection and re-entry. This approach enabled successful revascularization and demonstrated how enhanced image processing can directly influence procedural planning and efficiency. Importantly, total fluoroscopic and procedural times were 24.4 and 80 minutes, respectively; had we prematurely converted to a different strategy, these times may have been significantly higher.

## Conclusions

This case report illustrates how STEP can augment angiography in CTO PCI by clarifying lesion architecture and collateral circulation. STEP provided insights that were not apparent on the conventional angiogram, influencing strategy selection and facilitating procedural success. As adoption of machine learning–based imaging grows, such technologies may reduce the variability in lesion assessment, streamline planning, and improve outcomes in complex PCI.

## Funding Support and Author Disclosures

The authors have reported a research collaboration with AngioWave Imaging. The authors have reported that they have no relationships relevant to the contents of this paper to disclose.

## Appendix

Video 1 Diagnostic Cine, RAO 13°, CAU 36° (Unprocessed On Left, Processed on Right)

Video 2 Diagnostic Cine, RAO 2°, CAU 33° (Unprocessed on Left, Processed on Right)

Video 3 Diagnostic Cine, LAO 31°, CAU 33° (Unprocessed on Left, Processed on Right)

Video 4 Diagnostic Cine, LAO 27°, CRA 1° (Unprocessed on Left, Processed on Right)

Video 5 Final Angiography After CTO PCI of the Distal Circumflex Artery, RAO 11°, CAU 33° (Unprocessed Left, Processed Right)
